# Epidemiology of Chewing Lice (Phthiraptera: Mallophaga) Fauna of Poultry in Sub-Saharan Africa

**DOI:** 10.3390/pathogens14121192

**Published:** 2025-11-22

**Authors:** Silindokuhle Mlondo, Danisile Tembe, Mokgadi Pulane Malatji, Samson Mukaratirwa

**Affiliations:** 1 School of Agriculture and Science, College of Agriculture, Engineering and Science, University of KwaZulu-Natal, Durban 4000, South Africa; tembed@ukzn.ac.za (D.T.); pulanemalatji@gmail.com (M.P.M.); or smukaratirwa@rossvet.edu.kn (S.M.); 2 One Health Center for Zoonoses and Tropical Veterinary Medicine, Ross University School of Veterinary Medicine, Basseterre P.O. Box 334, Saint Kitts and Nevis

**Keywords:** chewing lice, biodiversity, poultry, epidemiology, distribution, prevalence, sub-Saharan Africa

## Abstract

Chewing lice are among the most significant ectoparasites affecting poultry, causing irritation, anemia, and reduced productivity, thereby posing economic and welfare challenges for poultry farmers. Their impact is particularly pronounced in sub-Saharan Africa, where poultry production is predominantly free-range with limited biosecurity, which increases exposure to infestation. This review was conducted to determine the epidemiology of chewing lice species of poultry in sub-Saharan African countries. A search of peer-reviewed literature on the epidemiology of chewing lice species of poultry was conducted on four electronic databases from 1990 to 2024. Nineteen species of chewing lice, namely *Menacanthus stramineus*, *Menacanthus cornutus*, *Menacanthus pallidulus*, *Menopon gallinae*, *Lipeurus caponis*, *Lipeurus tropicalis*, *Gallacanthus cornutus*, *Goniocotes gigas*, *Goniocotes gallinae*, *Goniocotes hologaster*, *Goniodes gigas*, *Goniodes meleagridis*, *Goniodes gallinae*, *Goniodes dissimilis*, *Cuclotogaster heterographus*, *Stenocrotaphus gigas*, *Columbicola columbae*, *Chelopistes meleagridis*, and *Amyrsidea powelli*, were reported from six poultry species distributed across ten African countries. The identification of the chewing lice species was primarily based on microscopic examination of the morphological features, which resulted in the exclusion of some studies that failed to identify lice to the species level. Poultry species infested included chickens (*Gallus gallus domesticus*), turkeys (*Meleagris gallopavo*), ducks (*Anas platyrhynchos*), guinea fowls (*Numida meleagridis*), pigeons (*Columba livia*), and geese (*Anser cygnoides*). Nigeria recorded the highest number of chewing lice species. The genus *Goniodes* showed the highest species diversity, and *M. stramineus* was the most predominant species, reported in nine of the ten reviewed countries. Infestations were mostly reported in chickens compared to other poultry species, and the prevalence ranged from 1.28% in chickens in Ethiopia to 100% in chickens from Zimbabwe. Results from this review provide valuable insights into the species diversity and regional distribution patterns of chewing lice fauna, highlighting their dispersion and host associations. The review will serve as a valuable resource in the design of effective and sustainable prevention and control strategies of chewing lice, especially in free-range chickens reared by resource-poor communities in sub-Saharan Africa.

## 1. Introduction

Poultry farming plays a significant economic role, which varies widely in both developed and developing countries. In developed countries, production has become highly specialized and integrated into a dynamic industry that is significantly contributing to food security globally [1]. However, in developing countries, poultry production is facing significant pressure to meet the rising demand for animal protein driven by population growth, while also aiming to generate surplus for international trade [2]. The poultry production industry faces significant challenges due to endo- and ectoparasites infestation, which negatively impact production [3]. Ectoparasites can directly impact poultry health by causing irritation, discomfort, and tissue damage, leading to anemia, allergic reactions, toxicosis, and dermatitis [4]. These negative effects not only affect the well-being of the birds but also reduce the quality and quantity of both meat and eggs [5,6].

As poultry production gains in significance, pathogens that compromise efficient production and animal welfare are increasingly becoming important [7]. According to [2,5,8], ectoparasitism accounts for the highest economic losses, especially to subsistence and small-scale farmers. Furthermore, they also play a significant role in the transmission of several pathogens, and exhibit host specificity due to adaptations to their host’s unique characteristics, such as body size, feather structure, and skin properties [9,10].

In sub-Saharan Africa, most poultry are reared under traditional husbandry practices, such as free-range and backyard systems [11], which expose birds to a wide range of parasites due to limited biosecurity and contaminated environments. Ectoparasites that infest poultry mostly belong to the phylum Arthropoda and are classified into two main classes: Arachnida, which includes ticks and mites (Order Acarina), and Insecta, which comprises lice (Order: Phthiraptera) and fleas (Order: Siphonaptera) [12].

Chewing lice (Order: Phthiraptera) are among the most significant ectoparasites affecting both domestic and wild birds [13]. Numerous species have been documented across sub-Saharan Africa [14,15,16,17,18,19,20], and these species include *Menacanthus stramineus*, *Menopon gallinae*, *Lipeurus caponis*, and *Cuclotogaster heterographus* [6,21,22]. *Menacanthus stramineus* is considered the most pathogenic poultry louse, due to its ability to induce severe anaemia by puncturing small feathers and feeding on the blood that seeps from the wounds [23], and consequently, causing skin inflammation and formation of extensive scabs [24]. Despite the impact, this group of ectoparasites often remains overlooked, especially in most developing countries, contributing to the scanty information published in sub-Saharan Africa. Therefore, this study sought to review peer-reviewed articles on the epidemiology of chewing lice in sub-Saharan Africa published between 1990 and 2024.

## 2. Materials and Methods

### 2.1. Literature Search Strategy

**Figure 1:**
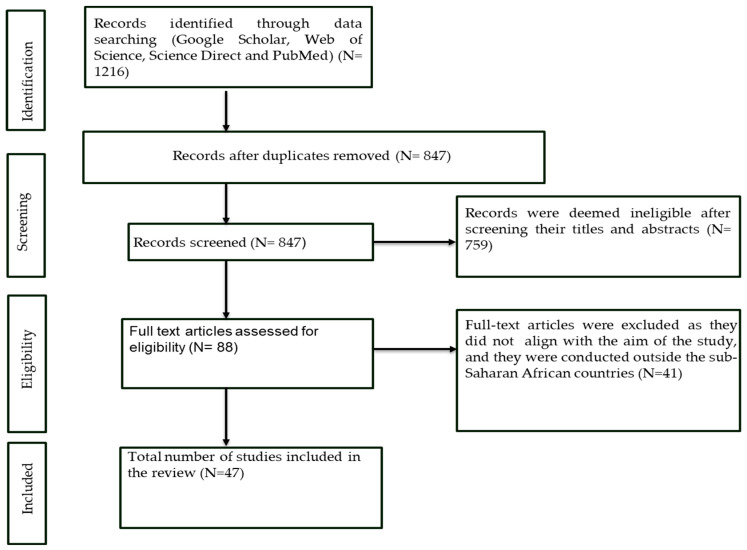
PRISMA diagram showing search and selection procedure.

A search of peer-reviewed literature was conducted on Google Scholar, Web of Science, Science Direct, and PubMed databases using Boolean operators (AND/OR) and a combination of the following search terms: “distribution” OR “occurrence “OR ‘’prevalence” OR “infestation” AND “chewing lice” OR “*Menacanthus*” OR “*Menopon*” OR “*Cuclotogaster*” OR “*Lipeurus*” OR “*Gallacanthus*” OR “*Goniodes*” OR “*Goniocotes*” OR “*Stenocrotaphus*” OR “*Columbicola*” OR “*Chelopistes*” OR “*Amyrsidea*” AND “poultry” OR “chickens (*Gallus gallus domesticus*)” OR “guinea fowls (*Numida meleagris*)” OR “ducks (*Anas platyrhyncos*)” OR “turkeys” (*Meleagris gallopavo*)” OR “pigeons (*Columba livia*)” OR “pheasants (*Phasianus colchicus*)” OR “quails (*Cortunix coturnix*)” OR “ostrich (*Struthio camelus*/*molybdophanes*)” in sub-Saharan African countries including Angola, Ethiopia, Nigeria, and all others. This search strategy was conducted following the guidelines of the Preferred Reporting Items for Systematic Reviews and Meta-Analysis (PRISMA) statement (Figure 1), using a protocol registered in the International Prospective Register of systematic reviews (PROPSPERO) (ID: 1218806).

The search and initial screening of duplicates, abstracts, and titles were conducted by S. Mlondo, and the data were validated by D. Tembe. Bibliographies of relevant studies were screened to identify additional relevant articles. Full texts were retrieved and managed on EndNote reference manager version x9 (Clarivate Analytics, Philadelphia, PA, USA).

### 2.2. Article Selection Process and Criteria Used

The following inclusion criteria were predetermined and used to appraise studies: (i) only peer-reviewed articles conducted and published between 1990 and 2024 were selected, (ii) research articles selected which reported on the prevalence and distribution of chewing lice in sub-Saharan Africa, (iii) chewing lice were identified to species level, (iv) studies were conducted within the geographic region of sub-Saharan Africa, and (v) published in English. Articles were excluded if they did not contain information contributing towards answering the scoping review questions and/or did not meet the inclusion criteria.

### 2.3. Charting the Data and Summarizing the Results

The following data was extracted from articles which met the inclusion criteria and recorded on Microsoft Excel spreadsheet: the author(s) information, aim(s) of the study, the country the studies were conducted from, the poultry species studied, number of poultry screened, chewing lice species identified, the prevalence of each louse species, and the identification technique(s) used. In cases where prevalence was not mentioned, it was calculated from the study by calculating the number of birds screened versus the number positive for a specific species, multiplied by 100. Where one study reported infection in more than one host species, or using more than one diagnostic test, the prevalence of infection data was recorded separately.

## 3. Results

A total of 1216 records were retrieved from Google Scholar, Web of Science, Science Direct, and PubMed (Figure 1). After removing 369 duplicates, 847 articles were screened for eligibility, and 759 articles were deemed ineligible and excluded. Full texts of 88 articles were assessed for eligibility using the predetermined inclusion criteria, and 41 articles did not meet the eligibility criteria.

**Figure 2:**
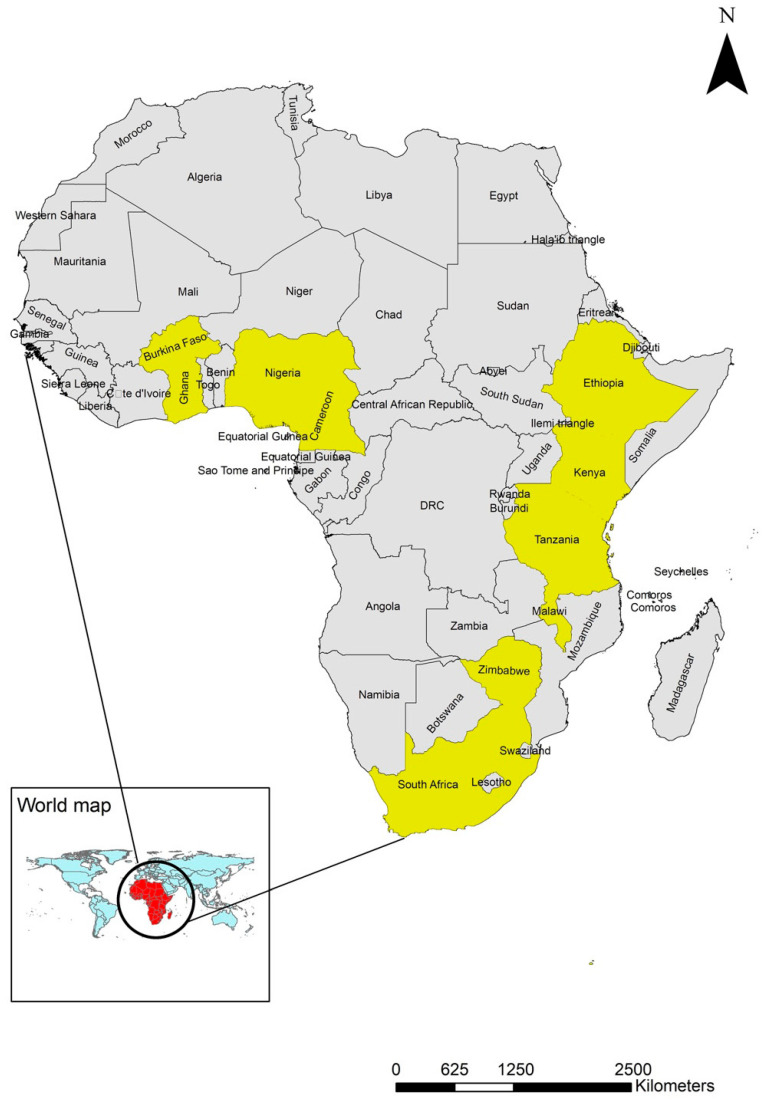
Map showing the ten sub-Saharan countries in yellow (Burkina Faso, Cameroon, Ethiopia, Ghana, Kenya, Malawi, Nigeria, South Africa, Tanzania, and Zimbabwe) with reports of ectoparasites in poultry (1990–2024).

**Figure 3:**
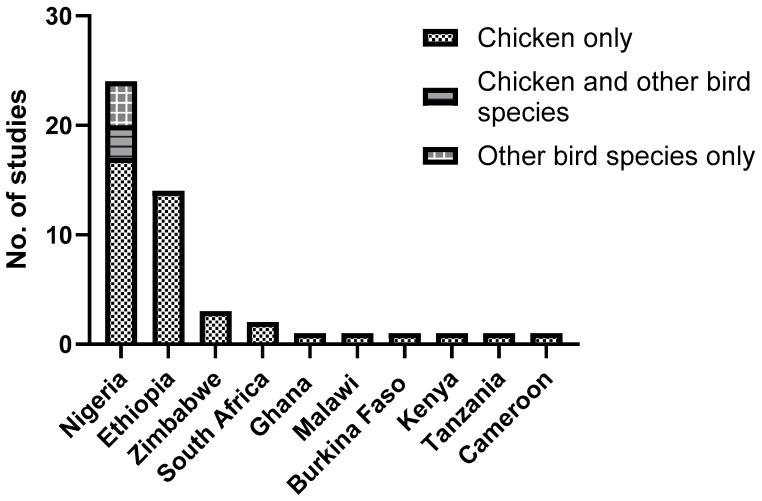
Number of studies reporting on chewing lice of poultry by country in sub-Saharan Africa (1990–2024).

The remaining forty-seven articles that met the inclusion criteria were reviewed, of which 49% (23/47) of these were conducted in Nigeria, 28% (13/47) in Ethiopia, 6% (3/47) in Zimbabwe, and 4% (2/47) in South Africa. Burkina Faso, Kenya, Tanzania, Cameroon, Ghana, and Malawi contributed 2% (1/47) each (Table 1, Figure 2). Thirty-nine studies documented infestations in chickens, and two in turkeys and pigeons each. Four studies investigated infestations in multiple poultry species (Figure 3).

### 3.1. Geographical Distribution and Species Diversity of Chewing Lice in Poultry

A total of 19 chewing lice species were identified as *Menacanthus* (*M.*) *stramenius*, *M. cornutus*, *M*. *pallidulus*, *Menopon* (*Me.*) *gallinae, Lipeurus* (*L.*) *caponis*, *L. tropicalis*, *Gallacanthus* (*Ga.*) *cornutus*, *Goniocotes* (*Go.*) *gigas*, *Go. gallinae*, *Go. hologaster*, *Goniodes* (*G.*) *gigas*, *G. meleagridis*, *G. gallinae*, *G.* *dissimilis*, *Cuclotogaster* (*C.*) *heterographus*, *Stenocrotaphus* (*S.*) *gigas*, *Columbicola* (*Co.*) *columbae*, *Chelopistes* (*Ch.*) *meleagridis*, *and Amyrsidea* (*A.*) *powelli* were recorded infesting poultry across ten sub-Saharan African countries from 1990 to 2024 (Table 1, Supplementary Table S1). The highest species diversity was noted in Nigeria, which recorded 13 of the 19 species documented, followed by South Africa (8/19), and Ghana reported the least number of chewing lice species, with only *Me. gallinae* and *M. stramineus* documented. *Lipeurus caponis*, *G. gigas*, and *Co. columbae* infested the highest number of poultry species.

Among the species documented, *M. stramenius* and *Me. gallinae* were the most common and widely distributed species. *Menacanthus stramineus* was documented in nine out of ten countries, namely Nigeria (chickens, turkeys), Ethiopia (chickens), Kenya (chickens), South Africa (chickens), Ghana (chickens), Malawi (chickens), Burkina Faso (chickens), Zimbabwe (chickens), and Cameroon (chickens). *Menopon gallinae*, which was the second distributed, was recorded in Nigeria (chickens, pigeons, ducks), Ethiopia (chickens), Tanzania (chickens), South Africa (chickens), Ghana (chickens), Burkina Faso (chickens), Zimbabwe (chickens), and Cameroon (chickens). *Lipeurus caponis* was identified in Nigeria (chickens, ducks, guinea fowls, pigeons, turkeys), Ethiopia (chickens), South Africa (chickens), Malawi (chickens), Burkina Faso (chickens, guinea fowls), and Zimbabwe (chickens).

Several species were restricted to a few countries, such as *M. cornutus*, *M. pallidulus*, and *A. powelli*, which were found exclusively in Nigeria, parasitizing only chickens. *Gallacanthus cornutus* and *S. gigas* were exclusively recorded in South African chickens. Similarly, *G. meleagridis* was found in Ethiopia in chickens. *Goniocotes gigas* infestations in chickens were documented in Ethiopia and South Africa. *Goniodes gigas* was recorded in Nigeria, Ethiopia, Tanzania, South Africa, Burkina Faso, and Zimbabwe, infesting chickens, pigeons, ducks, turkeys, and guinea fowls. *Goniodes gallinae* was recorded in Nigeria, Ethiopia, Burkina Faso, Zimbabwe, and Cameroon, affecting chickens, pigeons, and ducks. *Goniodes dissimilis* was found in Nigeria and Ethiopia, affecting chickens, pigeons, and ducks. Similarly, *C. heterographus* was reported in Nigeria, Ethiopia, and South Africa, infesting chickens, pigeons, and ducks. *Columbicola columbae* was recorded in Nigeria and Burkina Faso, parasitizing pigeons, chickens, ducks, turkeys, and guinea fowls. *Lipeurus tropicalis* was reported only in Nigeria, affecting chickens and turkeys, while *Ch. meleagridis* was also recorded in Nigeria only but infested chickens, pigeons, ducks, and turkeys.

### 3.2. Prevalence of Chewing Lice

Prevalence of chewing lice species in poultry across ten sub-Saharan African countries varied greatly, ranging from 1.28% to 100% (Table 2). The lowest prevalence of 1.28% (*n* = 390) was recorded in chickens infected with *M. stramineus* in Ethiopia [2], while the highest prevalence observed was 100% in chickens infected with *M. cornutus* (*n* = 100) [53], *Go. hologaster* (*n* = 13) in Nigeria [45], *Me. gallinae* (*n* = 18) in South Africa [17], and *M. stramineus* (*n* = 50) in Zimbabwe [16]. *Menopon gallinae*, *which* was the most frequently detected species infesting chickens, pigeons, and ducks, showed prevalence ranging from 4.5% (*n* = 200) in chickens [32] to 83.3% (*n* = 6) in ducks, both in Nigeria [45]. *Menacanthus stramineus* also exhibited a wide distribution, with prevalence ranging from 1.5% (*n* = 200) in chickens in Nigeria [32] to 100% (*n* = 50) in chickens in Zimbabwe [16].

Studies showed variation in the prevalence of chewing lice between and within countries. Ethiopia recorded the lowest prevalence of 5.2% (*n* = 384) in chickens infested with *Me. gallinae*, followed by *M. stramineus* with a prevalence of 7.8%(*n* = 384) [4]. The highest prevalence in this country was recorded with *Me. gallinae* and *L. caponis*, with a prevalence of 54.29% (*n* = 70) [49] and 19.5% (*n* = 384) [4], respectively. Similar observations were made in Nigeria, where *M. stramineus and Me. gallinae* recorded the lowest prevalence of 1.5% and 4.5% [32] in chickens (*n* = 200). *Menacanthus cornutus* also recorded the highest prevalence of 100% [53,54] in chickens (*n* = 100), followed by *L. tropicalis* with 78% in turkeys (*n* = 265) [19], and *Go. gallinae* with 74% also in chickens (*n* = 100) [53]. Kenya recorded a prevalence of 71.4% for *M. stramineus* in chicken [55]. *Menopon gallinae* demonstrated the highest prevalence in chickens in Malawi (34%), followed by *M. stramineus* (32%) [39].

In Zimbabwe, *M. stramineus* had the highest prevalence (100%, n = 50) while *Me. gallinae* was observed at 66% [16,52]. *Lipeurus caponis* had a low prevalence of 2% (*n* = 50) [51] and *Go. gallinae* was reported at 22% (*n* = 50) [51]. South Africa also recorded a prevalence (100%, *n* =18) in chickens infested with *Me. gallinae* [17], followed by *Go. gallinae* with 55.6% (*n* = 18) [17], while the lowest prevalence was reported with *L. caponis* (16.7%, *n* = 18) in chickens, and *G. gigas* 33.3% (*n* = 18) in chickens [17].

In Cameroon, *M. stramineus* had a prevalence of 16% (*n* = 400), while *Me. gallinae* was recorded at 26.3% (*n* = 400) [31]. *Goniocotes gallinae* in Cameroon had a lower prevalence of 4.5%(*n* = 400) [31]. In Tanzania, *Me. gallinae* had a prevalence of 48.6%, while *G. gigas* was recorded at 5.8% (*n* = 144) [14]. In Ghana, *Me. gallinae* and *M. stramineus* had a prevalence of 100% (*n* = 500) in chickens [5]. In Burkina Faso, chewing lice infestations were noted in chickens and guinea fowls, with *Me. gallinae* being the most commonly reported species [20].

### 3.3. Risk Factors Influencing the Distribution of Chewing Lice in Poultry

#### 3.3.1. Age of the Birds

The prevalence of chewing lice infestation was consistently higher in adults compared to young birds across multiple studies [1,14,49]. For instance, Lawal et al. [26] reported a prevalence of 61.75% in adult chickens and 22.75% in young chickens in Nigeria, and Maru et al. [50] documented a prevalence of 84.9% in adults compared to only 10.6% in young chickens in Ethiopia. Similar trends were documented by Zeryehun and Yohannes [29], Alemu et al. [42], and Mata et al. [6], indicating a significantly higher risk in adult birds in Ethiopia. Rabana et al. [30] further observed that adult turkeys were infested with chewing lice, with the prevalence of 44.0% as compared to 17.67% in young turkeys in Nigeria. However, Tamiru et al. [2] and Tessema et al. [11] observed a higher prevalence in younger chickens (74.45% and 41.92%) compared to adult chickens (61.79% and 13.66%), respectively, in Ethiopia.

#### 3.3.2. Sex of the Birds

Infestation of chewing lice was, in general, frequently higher in females compared to males. These observations were made by Malau and Rugu [33], who reported a prevalence of 61.18% in females compared to 38.82% in males in Nigeria. Abubakar and Aliyu [32] later reported the same trend of higher prevalence of 4.5% in females than the 2% observed in males, and Luka et al. [28] reported a prevalence of 46% in females compared to 30.46% in males in Nigeria. Alemu et al. [42] observed a slightly higher infestation in females (88.57%) than in males (87.14%), while Endale et al. [49] recorded a prevalence of 25.87% in females and 14.88% in males in Ethiopia. Other Ethiopian authors [50] reported a prevalence of 93.20% in females compared to 6.80% in males, and similar trends were documented by Mata et al. [6] and Kebede et al. [8]. In contrast, Tamiru et al. [2] and Amede et al. [4] reported results with higher infection rates in males (83.89% and 37.3%) than females (58.09% and 34.0%) birds in Ethiopia, respectively.

#### 3.3.3. Breed of the Birds

Endale et al. [49] and Maru et al. [50] recorded a higher prevalence of chewing lice in local poultry breeds (24.12% and 80.40%, respectively) compared to exotic breeds (19.08% and 19.90%, respectively). Similar observations were recorded by Tamiru et al. [2] and Mata et al. [6] in chickens in Ethiopia. Conversely, Alemu et al. [42] reported a higher prevalence in exotic breeds (98%) than in local ones (82.22%) in Ethiopia.

#### 3.3.4. Husbandry Practices

Studies showed that husbandry practices played a critical role in infestation rates of chewing lice. Birds reared under extensive husbandry systems showed higher prevalence rates across most studies. For instance, Lawal et al. [26] and Maru et al. [50] reported 64.75% and 83.90% in extensive systems, respectively, compared to significantly lower rates (16.67%) in semi-intensive or intensive setups [8]. Moreover, Shitta et al. [1] found a prevalence of 53.33% in free-ranging chickens compared to 31.50% in penned birds, but Tamiru et al. [2] observed an exceptionally high rate of 87.46% under an extensive husbandry system.

## 4. Discussion

Nineteen chewing lice species of poultry were identified across ten countries in sub-Saharan Africa. Among these, *Me. gallinae* and *M. stramineus* were the most widely distributed and recorded in almost all the studied countries. This widespread occurrence aligns with previous studies, indicating that these two species are the most common ectoparasites in poultry in Africa [16,43,55]. Furthermore, Nigeria recorded the highest chewing lice species diversity, compared to Ghana, which documented only *Me. gallinae* and *M. stramineus*, the most distributed species. Nahal et al. [56] also reported high species diversity with five species of chewing lice in chickens in northeastern Algeria, North Africa. In contrast, a recent study by Mohammed et al. [57] in chickens in Iraq reported only two species, *L. caponis* and *M. stramineus*, highlighting a comparatively lower species diversity, further highlighting the spread of the latter species. While the large number of studies from Nigeria may have caused bias in species diversity in this study, the remarkably high species diversity reported in the country compared to others can also be attributed to a combination of additional factors, including differences in production systems and environmental conditions [43,45,53]. Furthermore, the studies also highlighted that the distribution of these species is facilitated by infestation in chicken, as *L. caponis*, *G. gigas*, and *Co. columbae* were reported to infest the highest number of poultry species but have a limited distribution comparatively.

Several studies reported high prevalence of lice species in chicken, including *M. cornutus* in Nigeria [53], *Go. hologaster* in Nigeria [45], *Me. gallinae* in South Africa [17], and *M. stramineus* in Zimbabwe [16]. According to [19], the exceptionally high prevalence in these cases may be linked to a lack of awareness, poor husbandry and biosecurity, lack of regular ectoparasite control measures, which are all common in rural poultry production. Conversely, a low prevalence of 1.4% was recorded in Malawian chicken infested with *L. caponis* [39], suggesting that lice infestations can be effectively controlled under certain management systems. Ghana also showed low cases of infestation with *M. gallinae*, *L. caponis*, and *M. stramineus* 8.4%, and *Go. gallinae* in chickens [5]. Furthermore, a combination of controlled housing, reduced poultry density, and possibly more awareness of ectoparasite management may have contributed to the lower prevalence reported in this country. This observation was also made in other African countries where low prevalence rates were associated with improved poultry husbandry and biosecurity measures [51]. However, the lower number of studies conducted and reported in Malawi and Ghana may have also contributed towards the underreporting and underestimation of ecto-parasites in this country.

The study confirms that among the poultry species, chickens were the commonly studied across all ten countries, and this may be attributed to their high demand as a source of protein. It was further observed that chickens were the frequently infested poultry species, and this may be due to low production inputs associated with extensive husbandry practices, which create favorable conditions for the transmission and spread of chewing lice [58,59,60]. Ground-laying behavior brings chickens into closer contact with contaminated surfaces and nesting areas, further increasing their risk of infection [49,61]. Additionally, physiological factors such as hormonal fluctuations, particularly during laying periods, may influence immune responses and make chickens more susceptible to parasitic infestations [62]. This was highlighted by the consistent high prevalence of chewing lice infestation in females as compared to males [14,29,42], and adult birds attributed to their prolonged direct contact and more developed plumage, which offers greater habitat for lice colonization and egg-laying compared with the less-feathered bodies of younger birds [28,31,33]. Chewing lice are obligatory ectoparasites, and all life stages are completed on the host, and the high risk of transmission occurs when the chickens are brooding at night with limited space and have direct contact with each other [63].

Ducks and pigeons showed similar high infestation rates, with *Me. gallinae* reaching 83.3% in ducks in Nigeria [45], and *Co. columbae* reaching 60% in pigeons in Nigeria [15]. Similarities in high infestation rates in Nigeria, Kenya, and Zimbabwe may be attributed to the dominance of free-range poultry production systems, which increase transmission of chewing lice, especially during night roosting, when bird density is high, promoting transmission of the parasites through direct contact [16,43]. Fabiyi et al. [19] attributed the exceptionally high prevalence to poor biosecurity, lack of regular ectoparasite control measures, and free-range rearing systems, which are common in rural poultry production. Furthermore, reviewed studies showed that the prevalence of chewing lice also differed between breeds within poultry species, with local breeds showing a higher prevalence of chewing lice (87.55%) compared to exotic breeds (26.4%) [2]. This may be attributed to the differences in the husbandry practices, with local breeds predominantly reared under an extensive/free-ranging system compared to exotic breeds.

Ethiopia, Cameroon, and Tanzania recorded moderate prevalence rates, possibly due to a combination of semi-intensive and free-range rearing systems. Tamiru et al. [2] also recorded no infestation of lice in birds kept under a semi-intensive system. Although this was linked to the presence of routine parasite control and proper housing, the lower number of studies conducted in these countries may have resulted in the underestimation of chewing lice infestation in poultry. Despite these differences in environmental conditions, temperature and humidity likely play a role in shaping the prevalence of chewing lice across sub-Saharan Africa. Zeryehun and Yohannes [29] also supported that high temperatures and humidity in tropical climates favor lice survival and reproduction, leading to higher infestation rates in some areas. Conversely, Permin et al. [51] highlighted that lice populations may be naturally regulated in regions with dry or temperate conditions, leading to lower infestation rates. Furthermore, socio-economic factors, including access to veterinary care, quality of poultry housing, and farmer education and awareness on ectoparasite prevention and control, significantly influence infestation rates. Hence, regions with limited access to veterinary services tend to experience higher infestation burdens due to a lack of effective treatment options [55].

## 5. Conclusions

This review recorded the presence of 19 chewing lice species in six poultry species throughout ten sub-Saharan African countries. Documentation of these ectoparasites in only 10 out of 48 sub-Saharan countries underscores the paucity of research on this important sub-group of ectoparasites of poultry, and the burden of the infestation has not been adequately quantified, especially in resource-poor livestock farmers. *Menacanthus stramineus* and *M. gallinae* were the most predominant species of the documented poultry species, and most infections and studies were in chickens. Most studies primarily used morphological features to identify the lice to the species level, which can sometimes lead to failure to identify to the species level or misidentification, and we hereby recommend complementing with molecular techniques to characterize chewing lice species and generating genetic data for the biodiversity of lice in Africa. This review serves as a valuable resource in developing effective and sustainable prevention and control strategies of chewing lice, especially in the resource-poor communities in sub-Saharan Africa, where poultry meat serves as one of the inexpensive and affordable sources of protein.

## Figures and Tables

**Table 1:** Studies reporting chewing lice species in sub-Saharan Africa (1990–2024).

Chewing Lice Species	Country of the Study	Host Studied	Authors
*Menacanthus stramineus*	Nigeria, Ethiopia, Kenya, South Africa, Ghana, Malawi, Burkina Faso, Zimbabwe, Cameroon	Chickens, turkeys	[2,5,16,18,21,25,26,27,28,29,30,31,32]
*Menacanthus pallidulus*	Nigeria	Chickens	[1]
*Menopon gallinae*	Nigeria, Ethiopia, Tanzania, South Africa, Ghana, Burkina Faso, Zimbabwe, Cameroon	Chickens, pigeons, ducks	[4,5,6,8,14,15,16,17,18,20,21,22,26,28,29,31,32,33,34,35,36,37,38,39,40,41,42,43,44,45,46,47,48,49,50]
*Lipeurus caponis*	Nigeria, Ethiopia, South Africa, Malawi, Burkina Faso, Zimbabwe	Chickens, pigeons, ducks, turkeys, guinea fowls	[2,4,6,8,11,17,18,20,21,22,25,26,28,34,35,36,39,40,41,43,44,45,46,47,50,51,52]
*Gallacanthus cornutus*	South Africa	Chickens	[18]
*Goniocotes gigas*	Ethiopia, South Africa	Chickens	[11,17,39,41,49,52]
*Goniodes gigas*	Nigeria, Ethiopia, Tanzania, South Africa, Burkina Faso, Zimbabwe	Chickens, pigeons, ducks, turkeys, guinea fowls	[14,16,17,20,28,34,35,36,43,45,51,53]
*Goniodes meleagridis*	Nigeria	Chickens	[52]
*Goniodes gallinae*	Nigeria	Chickens, pigeons, ducks	[40,48]
*Goniocotes gallinae*	Nigeria, Ethiopia, Burkina Faso, Zimbabwe, Cameroon	Chickens, pigeons, ducks	[21,29,38,40,46]
*Goniocotes hologester*	Zimbabwe, Nigeria	Chickens	[38,45]
*Cuclotogaster* *heterographus*	Nigeria, Ethiopia, South Africa	Chickens, pigeons, ducks	[1,2,6,8,17,29,37,45,50]
*Stenocrotaphus gigas*	South Africa	Chickens	[18]
*Goniodes dissimilis*	Nigeria, Ethiopia	Chickens, pigeons, ducks	[35,40,45]
*Columbicola columbae*	Nigeria, Burkina Faso	Pigeons, chickens, ducks, turkeys, guinea fowls	[15,20,28,40,45,48]
*Lipeurus tropicalis*	Nigeria	Chickens, turkeys	[30,53]
*Chelopistes meleagridis*	Nigeria	Chickens, pigeons, ducks, turkeys	[30,40,45]
*Amyrsidea powelli*	Nigeria	Chickens	[33,53]
*Menacanthus cornutus*	Nigeria	Chickens	[1,33,53,54]

**Table 2:** Prevalence of chewing lice species in poultry from sub-Saharan Africa between 1990 and 2024.

Country of Study	Chewing Lice Species	Host Studied	Type of Husbandry	Total Examined	Total Infected	Prevalence (%)	Identification Method	Author(s)
Ethiopia	*Menacanthus stramineus*	Chickens	Intensive	384	30	7.8	Morphology	[4]
Ethiopia	*Menopon gallinae*	Chickens	Intensive	384	20	5.2	Morphology	[4]
Ethiopia	*Lipeurus caponis*	Chickens	Intensive	384	75	19.5	Morphology	[4]
Ethiopia	*Menopon gallinae*	Chickens	Extensive	450	40	8.9	Morphology	[29]
Ethiopia	*Cuclotogaster* *heterographus*	Chickens	Extensive	450	40	8.9	Morphology	[29]
Ethiopia	*Menacanthus* *stramineus*	Chickens	Extensive	450	37	8.3	Morphology	[29]
Ethiopia	*Menopon gallinae*	Chickens	Extensive	70	38	54.29	Morphology	[49]
Ethiopia	*Menacanthus* *stramineus*	Chickens	Extensive	70	20	28.57	Morphology	[49]
Ethiopia	*Goniodes gigas*	Chickens	Extensive	70	8	11.43	Morphology	[49]
Ethiopia	*Goniocotes gallinae*	Chickens	Extensive	70	4	5.71	Morphology	[49]
Ethiopia	*Cuclotogaster* *heterographus*	Chickens	Extensive	390	195	50	Morphology	[2]
Ethiopia	*Lipeurus caponis*	Chickens	Extensive	390	24	6.15	Morphology	[2]
Ethiopia	*Menacanthus* *stramineus*	Chickens	Extensive	390	5	1.28	Morphology	[2]
Nigeria	*Menopon gallinae*	Pigeons	Extensive	30	17	56.7	Morphology	[15]
Nigeria	*Columbicola columbae*	Pigeons	Extensive	30	18	60	Morphology	[15]
Nigeria	*Lipeurus tropicalis*	Turkeys	Extensive	265	207	78	Morphology	[19]
Nigeria	*Menacanthus* *stramineus*	Turkeys	Extensive	265	126	48	Morphology	[19]
Nigeria	*Chelopistes meleagridis*	Turkeys	Extensive	265	87	33	Morphology	[19]
Nigeria	*Menopon gallinae*	Ducks	Extensive	6	5	83.3	Morphology	[45]
Nigeria	*Gonoicotes hologaster*	Chickens	Extensive	13	13	100	Morphology	[45]
Nigeria	*Menopon gallinae*	Chickens	Extensive	1025	513	50.0	Morphology	[43]
Nigeria	*Goniodes gigas*	Chickens	Extensive	1025	139	13.6	Morphology	[43]
Nigeria	*Lipeurus caponis*	Chickens	Extensive	1025	227	22.1	Morphology	[43]
Nigeria	*Menopon gallinae*	Chickens	Extensive	200	9	4.5	Morphology	[32]
Nigeria	*Menacanthus* *stramineus*	Chickens	Extensive	200	3	1.5	Morphology	[32]
Nigeria	*Amyrsidea powelli*	Chickens	Extensive	100	50	50	Morphology	[53]
Nigeria	*Goniocotes gallinae*	Chickens	Extensive	100	74	74	Morphology	[53]
Nigeria	*Goniodes gigas*	Chickens	Extensive	100	56	56	Morphology	[53]
Nigeria	*Lipeurus tropicalis*	Chickens	Extensive	100	94	94	Morphology	[53]
Nigeria	*Menacanthus cornutus*	Chickens	Extensive	100	100	100	Morphology	[53]
Nigeria	*Menacanthus cornutus*	Chickens	Extensive	240	204	85	Morphology	[54]
South Africa	*Menopon gallinae*	Chickens	Extensive	18	18	100	Morphology	[17]
South Africa	*Goniocotes gallinae*	Chickens	Extensive	18	10	55.6	Morphology	[17]
South Africa	*Lipeurus caponis*	Chickens	Extensive	18	3	16.7	Morphology	[17]
South Africa	*Goniodes gigas*	Chickens	Extensive	18	6	33.3	Morphology	[17]
Zimbabwe	*Menacanthus* *stramineus*	Chickens	Extensive	50	50	100	Morphology	[16]
Zimbabwe	*Menopon gallinae*	Chickens	Extensive	50	33	66	Morphology	[17]
Zimbabwe	*Lipeurus caponis*	Chickens	Extensive	50	1	2	Morphology	[51]
Zimbabwe	*Goniocotes gallinae*	Chickens	Extensive	50	11	22	Morphology	[51]
Zimbabwe	*Menacanthus* *stramineus*	Chickens	Extensive	50	44	88	Morphology	[51]
Zimbabwe	*Menopon gallinae*	Chickens	Extensive	50	33	66	Morphology	[51]
Cameroon	*Menacanthus* *stramineus*	Chickens	Extensive	400	64	16	Morphology	[31]
Cameroon	*Menopon gallinae*	Chickens	Extensive	400	105	26.3	Morphology	[31]
Cameroon	*Goniocotes gallinae*	Chickens	Extensive	400	18	4.5	Morphology	[31]
Kenya	*Menacanthus* *stramineus*	Chickens	Extensive	360	257	71.4	Morphology	[55]
Malawi	*Menopon gallinae*	Chickens	Extensive	291	99	34	Morphology	[39]
Malawi	*Menacanthus* *stramineus*	Chickens	Extensive	291	93	32	Morphology	[39]
Malawi	*Lipeurus caponis*	Chickens	Extensive	286	4	1.4	Morphology	[39]
Tanzania	*Menopon gallinae*	Chickens	Extensive	144	70	48.6	Morphology	[14]
Tanzania	*Goniodes gigas*	Chickens	Extensive	144	8	5.8	Morphology	[14]

## Data Availability

Not applicable.

## Supplementary Materials

The following supporting information can be downloaded at https://www.mdpi.com/article/10.3390/pathogens14121192/s1, Supplementary Table S1: Checklist of studies reporting chewing lice species in different poultry husbandries from sub-Saharan Africa (1990–2024).
