# Supplementary material for: Epidemiology of Chewing Lice (Phthiraptera: Mallophaga) Fauna of Poultry in Sub-Saharan Africa
Source: Pathogens. 2025 Nov 22;14(12):1192. doi: 10.3390/pathogens14121192 (PMC12736369; doi:10.3390/pathogens14121192)
Supplement: Supplementary file 1 [file pathogens-14-01192-s001.zip › pathogens-3957078-supplementary.pdf]

Supplementary Table S1. Checklist of studies reporting chewing lice species in different poultry husbandries from sub-Saharan Africa (1990-2024).

Reference	Aims/ Objectives	Country of study	Host	Husbandry	Chewing lice species	Outcomes
[5]	To assess ectoparasites infestation in chickens of poultry farms that use the intensive system of poultry in the Sunyani West District of Ghana.	Ghana	Chickens	Intensive	*Menopon gallinae, Menacanthus stramineus,*	-Two species of lice were recovered from the chickens.<br>- This study observed 100% lice infestation.
[32]	To investigate ectoparasitic infestations to enhance management practices and improve productivity.	Nigeria	Chickens	Extensive	*Menopon gallinae, Menacanthus stramineus,*	-Two species of lice were identified from chickens.<br>-Out of the 200 chickens examined, lice were the dominant ectoparasites (6%).
[15]	To determine the species composition and prevalence of ectoparasites and helminths of Speckled Pigeon in the Zaria area of northern Nigeria.	Nigeria	Pigeon	Extensive	*Menopon gallinae, Columbicola columbae*	-Of the 30 pigeons examined, 18 (60%) were infested with ectoparasites.<br>-Of the ectoparasites recovered, two species were lice.
[34]	To determine the prevalence of ectoparasites in village chickens and to evaluate the economic significance of the ectoparasites infestation in Nigeria.	Nigeria	Chickens	Extensive	*Menopon gallinae, Lipeurus caponis, Goniodes gigas*	-Of the 1025 chickens examined, 136 (13.3%) were infested with lice.<br>-*Menopon gallinae* had the highest prevalence of 50.0%, and *Goniodes gigas* had the least prevalence of 13.6%.
[42]	To determine the prevalence of lice and fleas infestation and to assess the effect of host related risk factors in backyard chickens in Bishoftu town.	Ethiopia	Chickens	Extensive	*Menacanthus stramineus, Menopon gallinae, Cuclotogaster heterographus*	-Of the total of 140 chickens examined, 69.28% (97/140) were found positive for lice infestation.<br>-Lice species identified included *M. stramenius* (33.57%), *M. gallinae* (20.71%), and *C. hetrographus* (15%)
[4]	To determine the prevalence of ectoparasites infestation and to assess the effect of host related factors in intensive poultry farm at	Ethiopia	Chickens	Intensive	*Menacanthus stramineus, Menopon gallinae, Cuclotogaster*	-The overall prevalence of lice infestation was 35.1% (135/284)<br>- *L. caponis* (55.6%) was the most prevalent of the identified lice species, followed by *M. stramineus*

	Haramaya University, eastern Ethiopia.				*heterographus, Lipeurus caponis*	(22.2%), *M. gallinae* (14.8%) whereas *C. heterographus* (7.4%) was the least prevalent species.
[35]	To identify the major ectoparasites of local scavenging chickens in three agro-climatic zones.	Ethiopia	Chickens	Extensive	*Cuclotogaster heterographus, Goniodes gigas, Goniodes dissmilis, Goniocotes gallinae, Lipeurus caponis, Menacanthus stramineus, Menopon gallinae*	-Of all the ectoparasites recovered *Menopon gallinae* occurred with an overall prevalence rate of 87.9%.<br>- Lice were one of the two major ectoparasites identified in all the three areas.
[36]	To determine the prevalence rates of ectoparasites and to identify the species of ectoparasites in backyard chickens in three agroecologies in East Shoa of Oromia.	Ethiopia	Chickens	Extensive	*Menopon gallinae, Menacanthus stramineus, Goniodes gigas, Goniocotes gallinae, Cuclotogaster heterographus Lipeurus caponis*	- Of the total 600 local chickens examined, 84.3% were found to harbor lice.<br>Among the identified lice species, *M. stra mineus* was the most prevalent (65.3%) species followed by *C. hetrographus* (25%) and *M. gallinae* (19.6%)
[54]	To investigate the prevalence and incidence of *Menacanthus cornutus* in Kano State	Nigeria	Chickens	Intensive	*Menacanthus cornutus, Gonoides gigas*	- Of all the 240 chickens examined, only two species of lice were recovered.<br>-*Menacanthus cornutus* had the high prevalence (85%) and Gonoides gigas had the least prevalence (15%)
[21]	To identify ectoparasites found on domestic chickens in four areas of Sokoto	Nigeria	Chickens	Extensive	*Menopon gallinae, Menacanthus stramineus, Lipeurus caponis, Goniodes gigas, Goniocotes gallinae*	Of all the 160 chickens examined, 44 (27.5%) harbored lice.<br>-Five species of lice were recovered, and *M.gallinae* was more frequent.

[39]	To finding out the different ectoparasites species and their levels of infestation in different seasons and localities.	Malawi	Chickens	Extensive	*Lipeurus caponis, Menopon gallinae, Menacanthus stramineus, Gonocoites hologester, Gonocoites gigas*	-Five species of lice were recovered from the chickens.<br>- Menopon gallinae had the highest prevalence (34%), and G. hologaster had the least prevalence (0.3%).
[27]	To survey the ectoparasites associated with free-range system domestic fowl. *Gallus gallus* domesticus in Amassoma, Bayelsa State, Nigeria.	Nigeria	Chickens	Extensive	*Menacanthus stramineus*	-Only one species of lice was recovered from the chickens.<br>Of the 20 chickens examined, 4 (20%) were infested with lice.
[18]	To examine and identify the currently occurring ectoparasites in the Mnisi area.	South Africa	Chickens	Extensive	*Menopon gallinae, Gallacanthus cornutus, Goniocotes gallinae, Lipeurus caponis, Stenocrotaphus gigas, Menacanthus stramineus*	-Lice were isolated in 85% of the villages investigate and six species of lice were recorded.<br>- *Menopon gallinae* was recovered in 77% of all investigated villages and thereby most abundant.
[38]	To determine endo and ecto-parasites in Matebeleland , North and South from free range chickens.	Zimbabwe	Chickens	Extensive	*Menopon gallinae, Menacanthus stramineus, Goniocotes gallinae, Gonoicotes hologaster*	-Four species of lice were documented.<br>- *Menopon gallinae* had the highest mean number in infected chickens
[40]	To study the ecto- and helminth parasites of domestic birds and their implication on protein availability to the people of Etsako communities	Nigeria	Chickens, Pigeons, Ducks	Extensive	*Lipeurus caponis, Columbicola columbae, Cuclotogaster heterographus, Chelopistes*	-Ten species of lice were found on the birds.<br>- *Menacanthus stramenius* had the highest prevalence of 60.6%.

					meleagridis, Goniodes dissimilis, Goniodes gigas, Menopon gallinae Goniocotes gallinae, Menacanthus stramineus	
[45]	To determine the prevalence of infection with ecto- and endo-parasites in chickens, pigeons, and ducks in three local government areas of Edo Estate	Nigeria	Chickens, Pigeons, Ducks	Extensive	*Lipeurus caponis, Goniodes gigas, Goniodes dissimilis, Columbicola columbae, Cuclotogaster heterographus, Chelopistes meleagridis*	-Nine species of lice were recovered from the birds.<br>-*Menopon gallinae* was the most prevalent species recovered from chickens (22.39%) and ducks (100%).
[25]	To determine the prevalence of ecto- and endo parasites of local chickens in Abeokuta.	Nigeria	Chickens	Extensive	*Menacanthus stramineus, Lipeurus caponis*	-Two species of ectoparasites were recovered from chickens, *Menacanthus stramineus* with a prevalence of 90.0% and *Lipeurus caponis* with a prevalence of 60.0% were recovered.<br>-All the chickens examined were infected with one or two species of lice.
[49]	To identify and estimate the prevalence of domestic poultry ectoparasites and the assessment of associated risk factors managed under the backyard system in the Boloso Sore district of Wolaita zone southern Ethiopia.	Ethiopia	Chickens	Extensive	*Menopon gallinae, Menacanthus stramineus, Goniocotes gallinae, Goniocotes gigas*	-Out of all the 322 chickens examined, 21.73% (70/322) were infested with lice.<br>-*Menopon gallinae* showed a high prevalence of 54.29%, while *Goniocotes gallinae* had a low prevalence of 4%.
[19]	To determine the fauna, prevalence and seasonal patterns of	Nigeria	Turkeys	Extensive	*Lipeurus tropicalis, Goniocotes gallinae,*	-Four species of lice were reported.

	infestations of ectoparasites on turkeys.				*Menacanthus stramineus, Chelopistes meleagridis*	-*Lipeurus tropicalis* had the highest prevalence of 78%, and *Chelopistes meleagridis* had the least prevalence of 33%
[8]	To identify species composition of ectoparasites poultry circulating in the area; and to assess the prevalence and associated risk factors of poultry parasites in and around Jimma.	Ethiopia	Chickens	Extensive and Intensive	*Lipeurus caponis, Menopon gallinae, Menacanthus stramineus, Cuclotogaster heterographus*	- Of the 384 chickens examined, 42.71% (164/ 384) harbored lice.<br>- Four species of lice were identified, *Lipeurus caponis* had a high prevalence of 18.75% and *Menacanthus stramineus* had the least prevalence of 4.95%.
[31]	To determine the prevalence of chewing lice in local chickens; and to identify the lesions caused by chewing lice on local chickens	Cameroon	Chickens	Extensive	*Menacanthus stramineus, Goniocotes gallinae, Menopon gallinae*	-Lice was detected in 33.3% (133/400) of chickens examined.<br>-*Menopon gallinae* had the highest prevalence of 26..3% (105/400) and *Goniocotes gallinae* had the least prevalence of 4.5% (18/400).
[43]	To determine the prevalence of ectoparasites in village chickens, to identify species of ectoparasites infesting village chickens, and to evaluate the economic significance of ectoparasites infestation in Gombe, North-eastern Nigeria.	Nigeria	Chickens	Extensive	*Menopon gallinae, Lipeurus caponis, Goniodes gigas*	-Lice (85.8%) were the most prevalent ectoparasite encountered.<br>- Three species of lice were detected, with *Menopon gallinae* (50.0%) having the highest prevalence rate , followed by *Lipeurus caponis*, and *Goniodes gigas* (13.6%) having the lowest prevalence rate.
[26]	To determine the prevalence of ectoparasites of village chickens and their associated risk factors in and around Potiskum Yobe State, North-eastern Nigeria.	Nigeria	Chickens	Extensive	*Menopon gallinae, Menacanthus stramineus, Lipeurus caponis,*	-Out of the 400 chickens examined, lice were most dominant ectoparasites detected, with a prevalence rate of 57%.<br>-The three species of lice recovered from chickens were *Lipeurus caponis* (40.25%) *Menacanthus stramineus* (14.0%) and *Menopon gallinae* (2.75%).

[44]	To determine the chewing lice species richness, lice abundance, composition, sex, ratio, and percent prevalence in selected poultry farms in Benin City, Edo State	Nigeria	Chickens		*Menopon gallinae, Menacanthus stramineus, Lipeurus caponis,*	-Three species of lice were encountered.<br>-*Menacanthus stramineus* had a high prevalence of 29.7% and *Lipeurus caponis* had a low prevalence of 10.9%
[33]	To determine the ectoparasites that infest chickens Bokkos local government area of Plateu State Nigeria, and to adopt ways of improving poultry management and control of ectoparasites infestation in order to improve healthy growth of the animal and increases egg and meat production of the community.	Nigeria	Chickens		*Menacanthus cornutus, Amyrsidea powelli*	-Two species of lice were detected.<br>- The prevalence rates of *Menacanthus cornutus* and *Amyrsidea powelli* were 48.14%.
[50]	To determine the prevalence and variety of chicken ectoparasites, as well as their preferred host sites and potential risk factors related to their infestation.	Ethiopia	Chickens	Extensive and semi-intensive	*Menopon gallinae, Lipeurus caponis, Cuclotogaster heterographus*	-Out of the 384 chickens examined, 49 (12.8%) were found positive for lice.<br>-Three species of lice were detected, Lipeurus caponis had a prevalence of (5.47%), followed by *Menopon gallinae* (4.43), and *Cuclogaster heterographus* had the least prevalence of 2.86%.
[6]	To identify species composition, assess prevalence and associated risk factor of ectoparasite of poultry in extensive and intensive farms in and around Jimma town.	Ethiopia	Chickens	Extensive and Intensive	*Menopon gallinae, Lipeurus caponis, Menacanthus stramineus, Cuclotogaster heterographus*	-Of the 384 chickens examined, 28% (108/384) were found positive with lice.<br>-*Lipeurus caponis* had a high prevalence of 14%, and *Menacanthus stramineus* had the least prevalence of 1.5%.

[14]	To determine the prevalence and risk factors of ectoparasite infestation in Morogoro Municipality.	Tanzania	Chickens	Extensive	*Menopon gallinae, Goniodes gigas*	-Two species of lice *Menopon gallinae* with a prevalence of 48.6% and *Goniodes gigas* with a prevalence of 5.8% were recorded.
[37]	To identify and estimate prevalence of ectoparasites of poultry in intensive and backyard chicken farm at Wolayta Soddo town.	Ethiopia	Chickens	Extensive	*Menopon gallinae, Menacanthus stramineus, Cuclotogaster heterographus, Goniodes gigas, Goniocotes gallinae*	-Five species of lice were recovered from the chickens.<br>- *Menopon gallinae* 49% (139/284) the most prevalent ectoparasite species followed by *Cuclotogaster heterographus* 40%(115/284).
[16]	To provide information on husbandry aspects of free-range chickens in selected rural districts of Zimbabwe and document the species of ectoparasites and cestodes affecting them.	Zimbabwe	Chickens	Extensive	*Menopon gallinae, Goniodes gigas, Menacanthus stramineus, Lipeurus caponis*	-Five species of lice were detected from the chickens.<br>-*Menacanthus stramenius* had the highest overall prevalence of 87.7%, and *Lipeurus caponis* had the least prevalence of 5.2%.
[17]	To identify the species of lice infesting free-range chickens from selected rural communities of KwaZulu-Natal province and to determine the prevalence and mean abundance of the species identified.	South Africa	Chickens	Extensive	*Menopon gallinae, Goniocotes gallinae, Goniodes gigas, Lipeurus caponis, Cuclotogaster heterographus*	-Five species of lice were identified.<br><br>- *Menopon gallinae* was the most prevalent species across all the four communities with a prevalence of 96.8%.
[41]	To determine the prevalence of ectoparasites in poultry managed in backyard production system and to identify the species of ectoparasites.	Ethiopia	Chickens	Extensive	*Menopon gallinea, Menacanthus stramineus, Lipeurus caponis, Goniocotes gigas, Goniocotes gallinae*	-Five species of lice were encountered.<br>- *Menopon gallinae* had a high prevalence of 44.95%, and Goniocotes gallinae had the least prevalence of 6.4%.

[55]	To describe the prevalences of parasitic infections and infestations in the free-range village chickens in Machakos District of Kenya.	Kenya	Chickens	Extensive	*Menacanthus stramineus*	-One species of lice was reported.<br>- *Menacanthus stramineus* had a high prevalence of 79.4%.
[22]	To provide more information on the ectoparasites and gastrointestinal helminths of pigeons in Awka, South-eastern Nigeria.	Nigeria	Pigeons	Extensive	*Menopon gallinae, Goniodes gallinae, Columbicola columbae*	-Three species of lice were recovered from the pigeons.<br>-Of the 30 pigeons examined, 10(33.33%) were infested with *Columbicola columbae while Gonoides gallinae* was found in 4 (13.33%) pigeons.
[48]	To identify the taxa of ectoparasitic arthropods that infest free-range domestic fowls (*Gallus domesticus*) in Amansea and Ifite communities in Awka capital Territory and to determine the prevalence of such parasites and their levels of infestation in different localities.	Nigeria	Chickens	Extensive	*Menopon gallinea, Lipeurus caponis, Goniocotes gallinae*	-A total of 41.07% of the 112 chickens examined were harboured lice.<br>-Three species of lice were detected from the chickens.
[52]	To determine the prevalence of ectoparasites of domestic chickens in Gwagwalada Area Council and compare the parasitic load between the domestic and exotic breeds of chickens	Nigeria	Chickens	Extensive	*Menacanthus stramineus, Lipeurus caponis, Goniocotes gigas*	-Three species of lice were recovered from the chickens.<br>-Out of 250 chickens examined, *Menacanthus stramineus* had a prevalence of 52.8% (132/250), followed by Goniocotes gigas with a prevalence of 50.4% (126/250). *Lipeurus caponis* had the least prevalence of 28.4% (71/250).
[46]	To identify ectoparasite species on chickens; and to determine their infestation prevalence, preferred sites and associated risk factors.	Nigeria	Chickens	Intensive	*Menopon gallinea, Menacanthus stramineus, Lipeurus*	-Four species of lice were detected from the chickens.<br>- *Menopon gallinae* had a prevalence of 18.6%, followed by *Menacanthus stramineus* (18%) and

					caponis, Goniocotes gallinae	*Lipeurus caponis* (10.4%). *Goniocotes gallinae* ad the least prevalence of 5.1%.
[47]	To identify species composition of ectoparasites of poultry in the study area; and to assess the prevalence and associated risk factors in three selected poultry farms in Awka	Nigeria	Chickens	Extensive	*Menopon gallinea, Menacanthus stramineus, Lipeurus caponis,*	-Three species of lice were recovered from the chickens.<br>- *Menacanthus stramineus* had a highest species composition of 33.3%.
[51]	To describe the prevalences of parasitic infections and infestations in free-range village chickens in the Goromonzi District in Zimbabwe.	Zimbabwe	Chickens	Extensive	*Menopon gallinea, Menacanthus stramineus, Lipeurus caponis, Goniocotes gallinae, Goniodes gigas*	-Five species of lice were detected from the chickens.<br>- *Menopon gallinae* was found in 90% of the young chickens examined, and in 88% of the adult chickens studied.
[28]	To determine the occurrence and species of ectoparasites on available domesticated poultry species in Maiduguri, Borno State and to comparatively determine their distribution across the various species.	Nigeria	Chickens, Turkeys, Guinea fowls, Pigeons, Geese, Ducks	Extensive	*Menopon gallinae, Menacanthus stramineus, Lipeurus caponis, Goniodes gigas, Goniocotes gallinae, Columbicola columbae*	- The highest infestation among the species of poultry examined was in chickens.<br>-*Lipeurus caponis* recovered was the most abundant, followed by *Menopon gallinae* and *Goniocotes gallinae,* while *Columbicola columbae* was the least prevalent parasite
[30]	To determine the prevalence rates of ectoparasites in domesticated turkeys in Jere area, Borno State, Nigeria.	Nigeria	Turkeys	Extensive	*Lipeurus tropicalis, Menacanthus stramineus, Chelopistes meleagridis,*	-Three species of lice were found on the body of the turkeys.<br>-*Lipeurus tropicalis* have a prevalence of 17.67%, followed by *Menacanthus stramineus* with a prevalence of 8.33%. *Chelopistes meleagridis* had the least prevalence of 5.67%
[1]	To identify available ectoparasites of chickens, and the ages and life-style of birds, in order to	Nigeria	Chickens	Extensive and Intensive	*Cuclotogaster heterographus, Menacanthus*	-Of the 500 chickens studied, 220 (44.00%) were infested with lice.

	recommend suitable methods by which poultry production can be improved through reduction in the ectoparasitic infestation of birds and to provide baseline information on ectoparasites of these important domestic birds in Wukari.				*stramineus, Menacanthus cornutus, Menacanthus pallidulus*	- *Cuclogaster heterographus* was detected in half of the chickens, *Lipeurus caponis* was present in 6.15% of them, and *Menacanthus stramineus* was identified in 1.28% of the chickens.
[2]	To determine the prevalence of chicken ectoparasites, to identify species of ectoparasites affecting chickens and to asses potential risk factors.	Ethiopia	Chickens	Extensive and Semi-intensive	*Cuclotogaster heterographus, Menacanthus stramineus, Lipeurus caponis*	-Three species of lice were detected from the body of chickens.<br>-
[29]	To determine the prevalence of parasites , to identify the fauna and the host related risk factors in free scavenging chickens in Wolayita Zone.	Ethiopia	Chickens	Extensive	*Cuclotogaster heterographus, Menacanthus stramineus, Menopon gallinae, Goniocotes gallinae*	-Out of the 450 chickens examined, 62 (13.8%) were infested with lice.<br>- Five species of lice were detected from the body of the chickens.
[11]	To determine the prevalence of ectoparasites in poultry managed in the backyard production system, and to identify the species of ectoparasites in poultry in Mareka Wodera.	Ethiopia	Chickens	Extensive	*Menopon gallinae, Menacanthus stramineus Lipeurus caponis, Goniocotes gallinae*	-Four species of lice were found on the body of the chickens.<br>-Of the 384 chickens examined, 33.85% (109/384) were positive for lice.
[53]	To determine the prevalence of ecto and haemoparasites of chickens in Sokoto metropolis.	Nigeria	Chickens	Extensive	*Amyrsidea powelli, Goniocotes gallinae, Goniodes gigas, Lipeurus tropicalis, Menacanthus cornutus*	-Five species of lice were detected from the chickens.<br>- *Menacanthus cornutus* had a high prevalence of 100% in chickens raised in a free-range production system while in chickens raised in an intensive, Menacanthus cornutus had a low prevalence of 50%.

[20]	To find information on the diversity of ectoparasites of hens and guinea fowls raised in an extensive system in the commune of Loumbila.	Burkina Faso	Chickens, Guinea fowls	Extensive and Intensive	*Menopon gallinae, Menacanthus stramineus, Goniodes gigas, Goniocotes gallinae, Lipeurus caponis, Columbicola columbae*	-Six species of lice were reported.<br><br>- *Menacanthus stramineus* was the most abundant species with a prevalence of 76.24% and was mostly found in chickens.
